# Supplementary figures and images for: The small acid-soluble proteins of Clostridioides difficile are important for UV resistance and serve as a check point for sporulation
Source: PLoS Pathog. 2021 Sep 8;17(9):e1009516. doi: 10.1371/journal.ppat.1009516 (PMC8452069; doi:10.1371/journal.ppat.1009516)

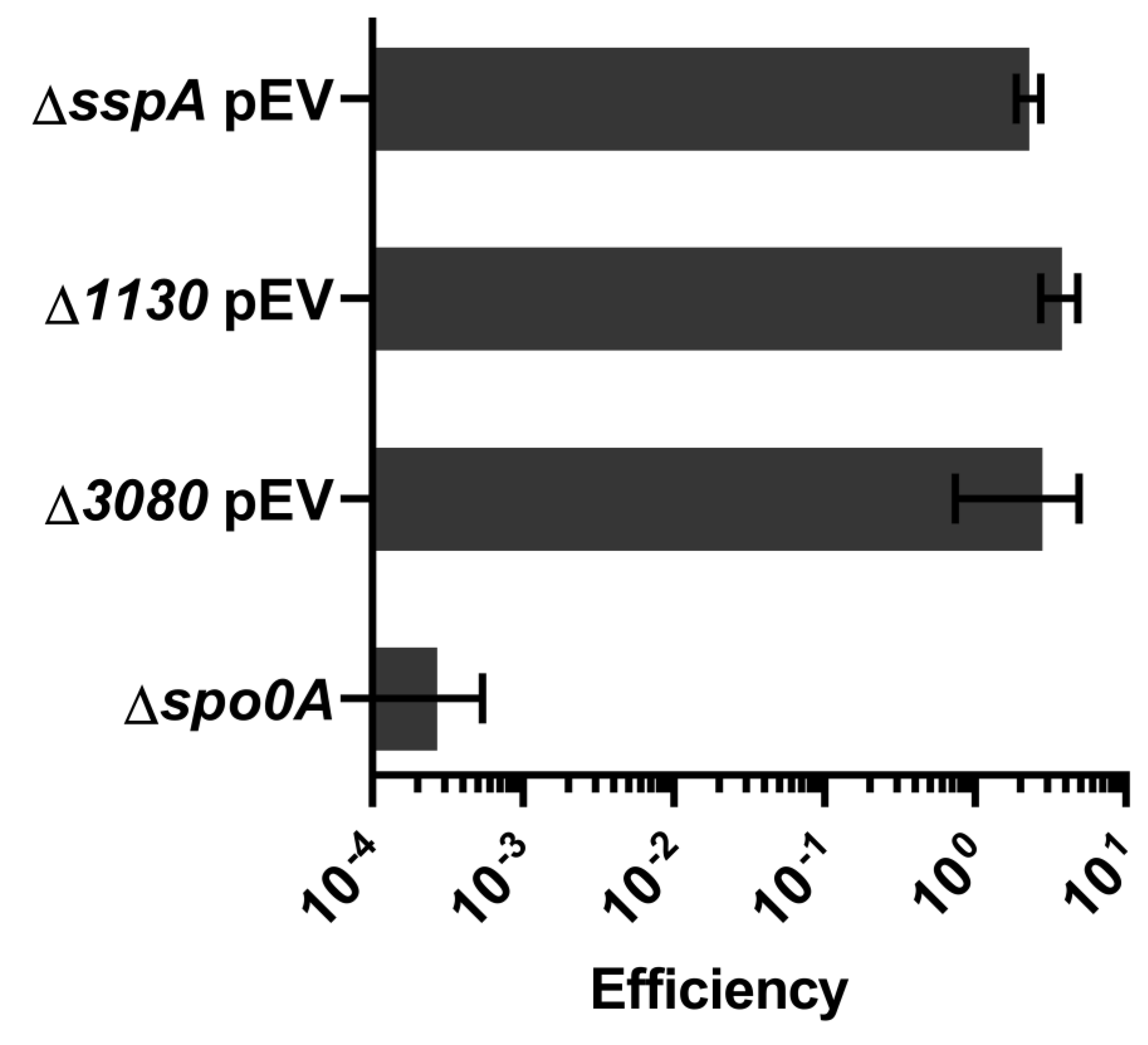

Supplement: S1 Fig — Strains were grown on sporulation medium for two days. Sporulating cultures were heat treated at 65°C. Sporulation rate was determined by comparison of the CFU of heat treated culture to CFU of untreated culture and then the ratios were compared to wildtype. pEV indicates an empty vector. All data represents the average of three independent experiments. Statistical analysis by one way ANOVA with Dunnett’s multiple comparison test. (TIF) [file ppat.1009516.s001.tif]

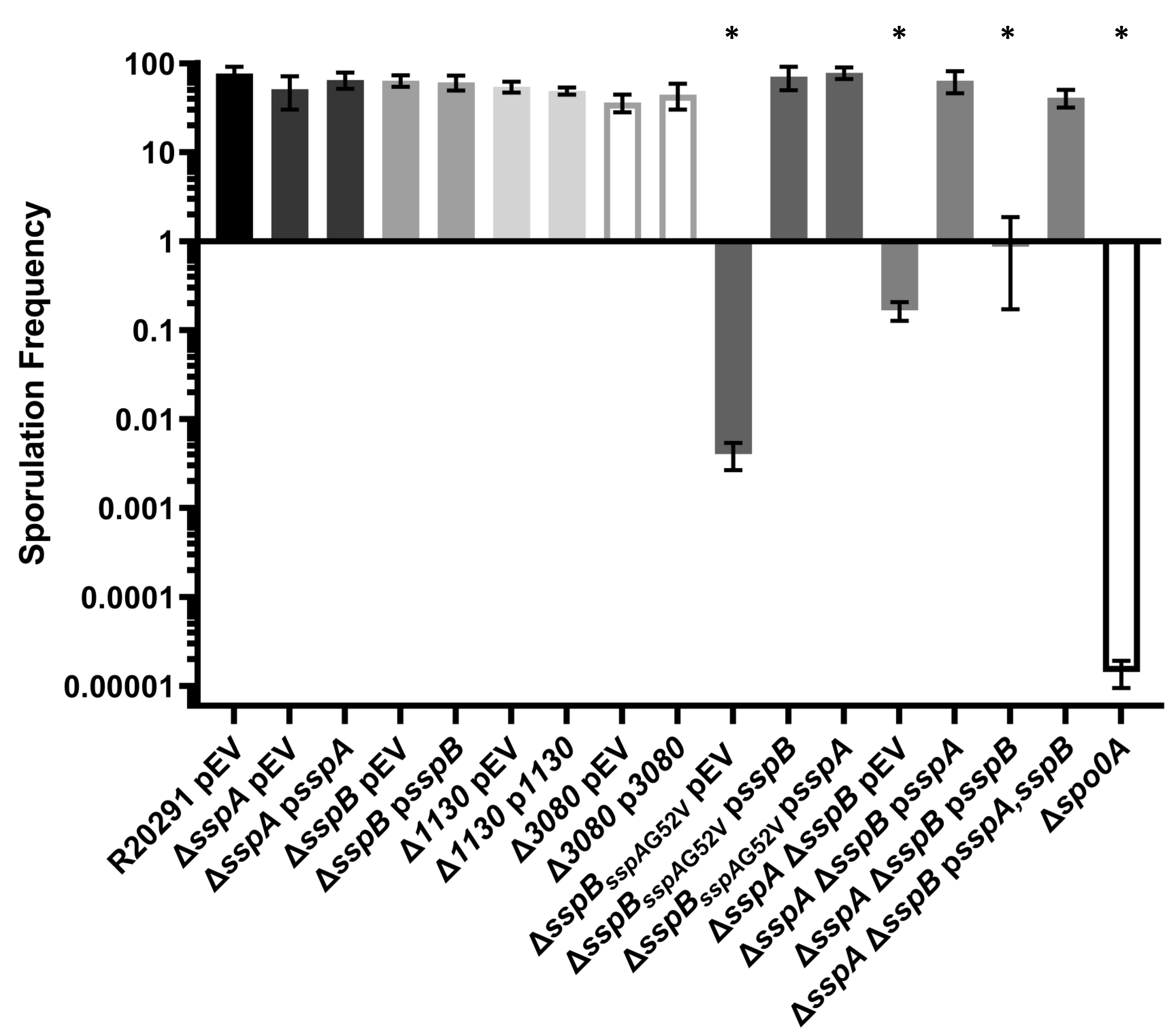

Supplement: S2 Fig — The strains were treated with 28.5% final concentration of ethanol. The sporulation frequency was determined by dividing the number of spores by the total number of cells (spores + vegetative cells). C. difficile Δspo0A was set to the limit of detection to determine sporulation frequency. All data represent the average of three independent experiments and the standard error from the mean. Statistical analysis by one way ANOVA comparison to wildtype with Dunnett’s multiple comparison test. * p < 0.001. (TIF) [file ppat.1009516.s002.tif]

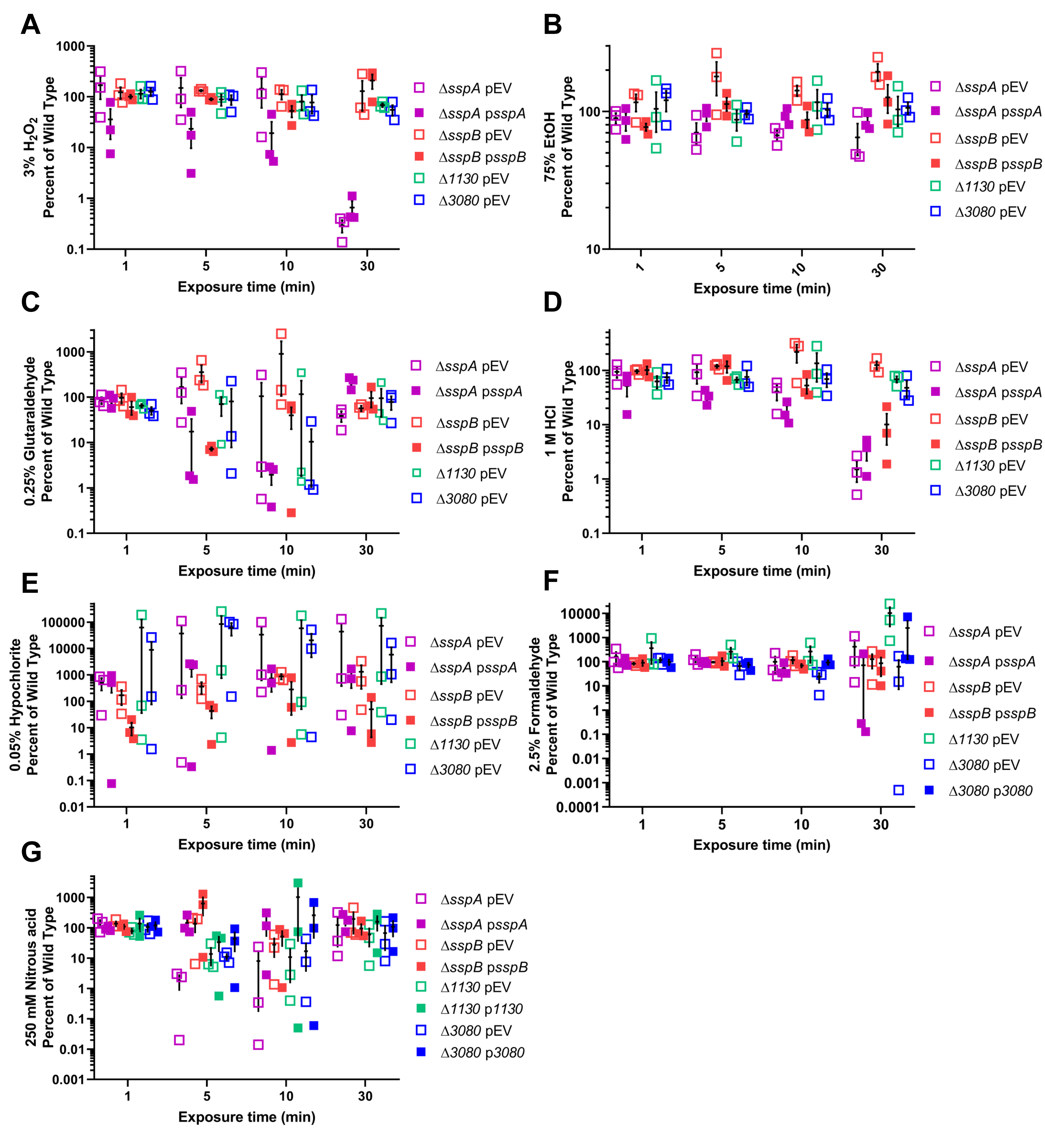

Supplement: S3 Fig — 1 x 10^9^ spores were exposed to chemicals for 1, 5, 10, or 30 minutes. After exposure, solutions were serially diluted and plated onto rich medium with germinants. The CFUs were enumerated and compared to unexposed samples and then this ratio was compared with that of the wildtype spores. A) 3% H202 B) 75% EtOH C) 0.25% Glutaraldehyde D) 1 M HCL E) 0.05% hypochlorite F) 2.5% Formaldehyde G) 250 mM Nitrous Acid. pEV indicates an empty vector. All data represents the average of three independent experiments. Statistical analysis by two way ANOVA with Dunnett’s multiple comparison. A) C. difficile ΔsspA pEV and C. difficile ΔsspA psspA P<0.0001 at 30 minutes. C) P<0.001 for C. difficile ΔsspB psspB 5 minutes and C. difficile ΔsspA psspA at 10 minutes. P<0.05 for C. difficile ΔCDR20291_3080 pEV at 10 minutes. D) P<0.05 for C. difficile ΔsspA psspA at 5 minutes and C. difficile ΔsspB psspB at 30 minutes. P<0.01 for C. difficile ΔsspA psspA at 10 minutes. P<0.001 for C. difficile ΔsspA pEV and C. difficile ΔsspA psspA at 30 minutes. E) P<0.01 for C. difficile ΔsspB psspB at 1 minute. F) P<0.05 for C. difficile ΔCDR20291_3080 at 10 minutes. G) P<0.05 for C. difficile ΔCDR20291_1130 at 5 minutes, C. difficile ΔsspA pEV and C. difficile ΔCDR20291_1130 pEV at 10 minutes. P<0.01 for C. difficile ΔCDR20291_3080 at 5 minutes. P<0.001 for C. difficile ΔsspA pEV at 5 minutes. (TIF) [file ppat.1009516.s003.tif]

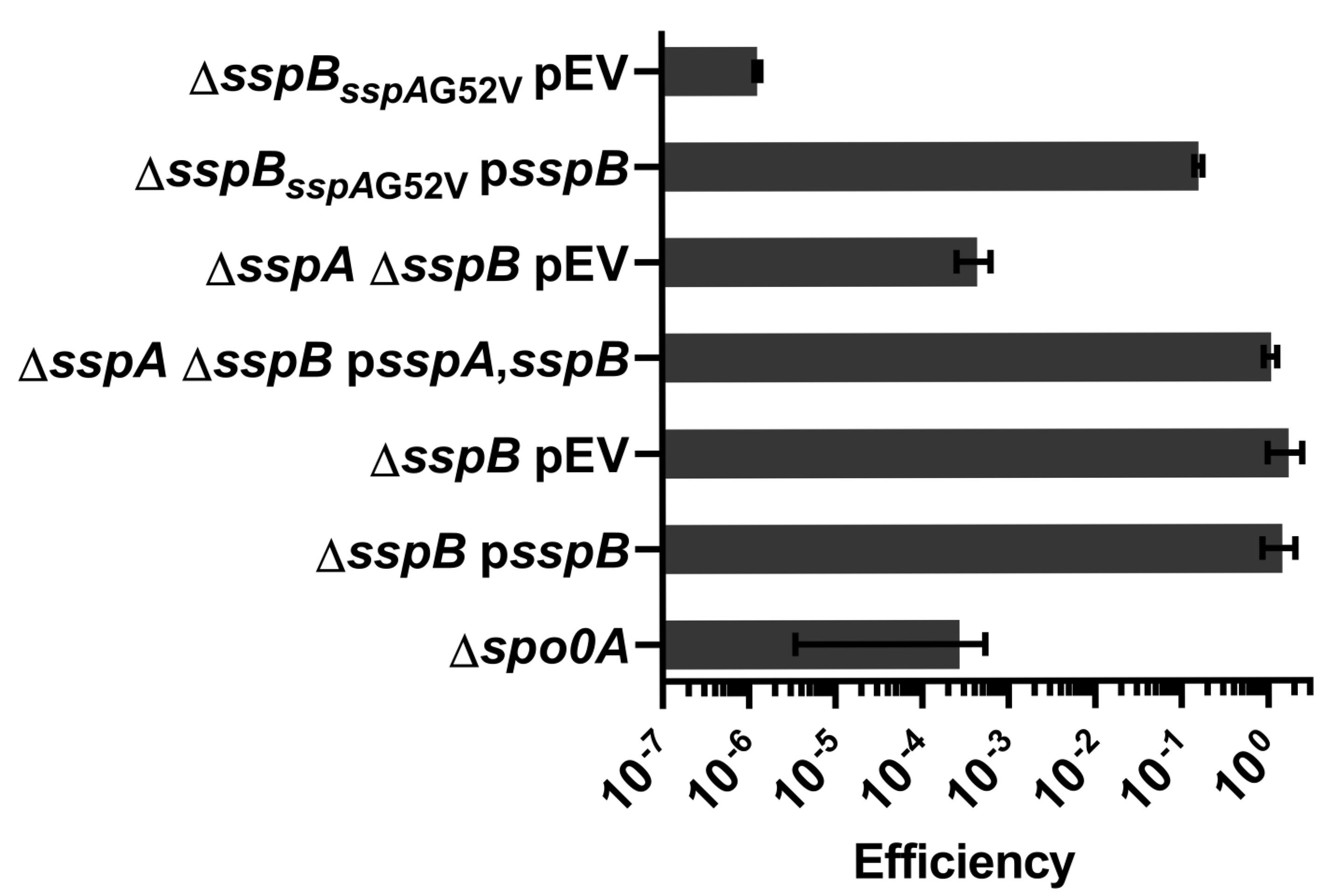

Supplement: S4 Fig — Strains were grown on sporulation medium for two days. Sporulating cultures were heat treated at 65°C. Sporulation rate was determined by taking the ratio of the CFU of heat treated culture to CFU of untreated culture and then the ratios were compared to wildtype. pEV indicates an empty vector. All data represents the average of three independent experiments. Statistical analysis by one way ANOVA with Dunnett’s multiple comparison test. (TIF) [file ppat.1009516.s004.tif]

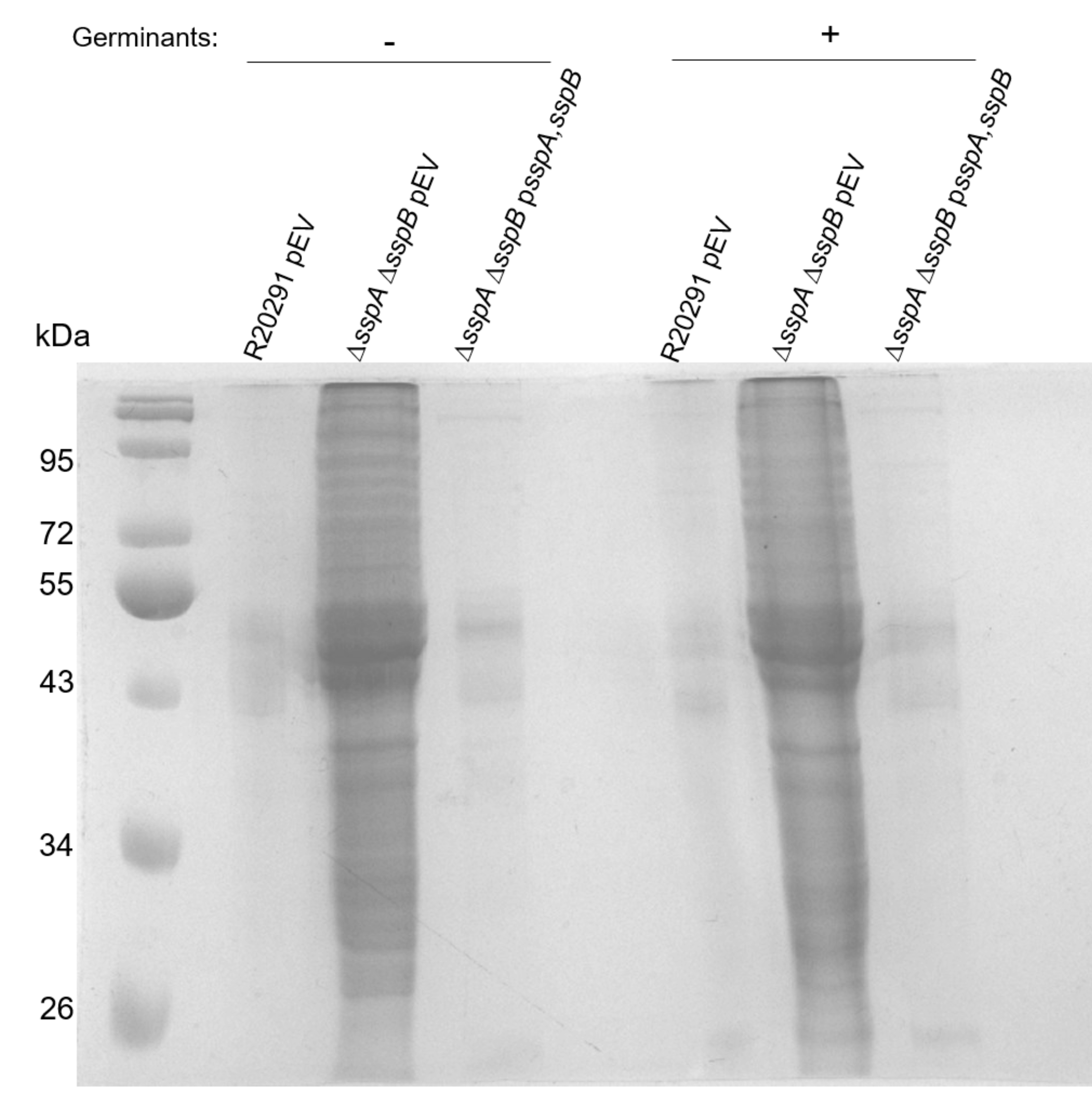

Supplement: S5 Fig — The same sample volumes used for the ΔsspA ΔsspB SleC cleavage assay were separated by a 15% SDS PAGE and stained with Coomassie. pEV indicates an empty vector. (TIF) [file ppat.1009516.s005.tif]

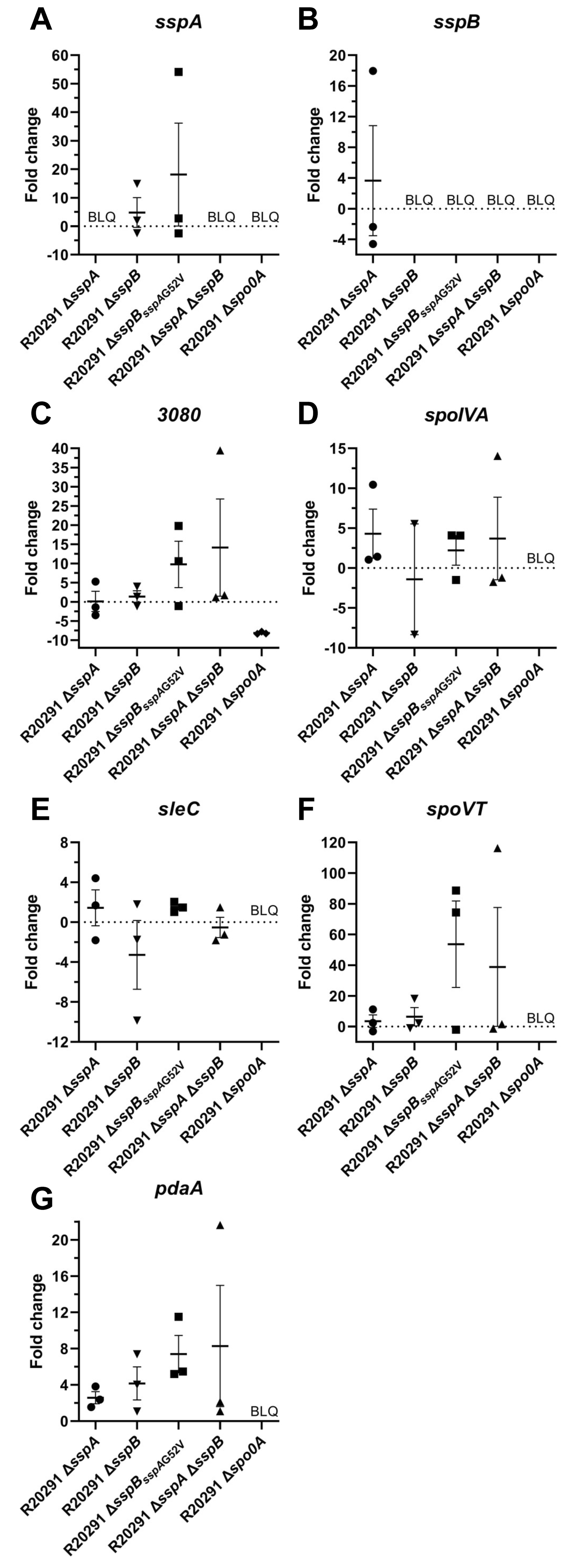

Supplement: S6 Fig — RNA was extracted from wildtype and mutant strains. DNA was depleted from the RNA samples and then cDNA was generated. Transcript levels were determined through quantitative reverse transcriptase PCR and the fold change between mutant transcript levels and wildtype levels was determined. A) RNA extracted after 48 hours of incubation, sspA transcript levels. B) RNA extracted after 48 hours of incubation, sspB transcript levels. C) RNA extracted after 48 hours of incubation, 3080 transcript levels. D) RNA extracted after 18 hours of incubation, spoIVA transcript levels. E) RNA extracted after 24 hours of incubation, sleC transcript levels. F) RNA extracted after 48 hours of incubation, spoVT transcript levels. G) RNA extracted after 48 hours of incubation, pdaA transcript levels. BLQ is below limit of quantification; the cycle threshold is below the limit of which we can accurately quantify. All data represents the average of three independent experiments and the standard error from the mean. Statistical analysis by one way ANOVA with Dunnett’s multiple comparison test. (TIF) [file ppat.1009516.s006.tif]
