# Supplementary material for: The small acid-soluble proteins of Clostridioides difficile are important for UV resistance and serve as a check point for sporulation
Source: PLoS Pathog. 2021 Sep 8;17(9):e1009516. doi: 10.1371/journal.ppat.1009516 (PMC8452069; doi:10.1371/journal.ppat.1009516)
Supplement: S1 Table — (DOCX) [file ppat.1009516.s007.docx]

**S1 Table. Primers used in this study.**

| **Primer Name** | **Sequence** |
| --- | --- |
| 5'sspA_MTL | ttatcaggaaacagctatgaccgcggccgcttagatgaggaaaaactggataa |
| 3'sspA_up | ttatttataactatctgttgctttttccaggttgattaccttccttctgttta |
| 5'sspA_down | aataaattaaacagaaggaaggtaatcaacctggaaaaagcaacagatagt |
| 3'sspA_downMTL | ctgcgatcgcgcatgtctgcaggcctcgagctattgaacttggaaatgagag |
| CRISPR_sspA_165 | gtgtgctataattaaactgtaaaacgcgtGACTAAAAAATTAGTTGAAAGTTTTAGAGCTAGAAATAGCAAGTTAAAATAAGGCTAGTCCGTTATCAACTTGAAAAAGTGGCACCGAGTCGGTGCTTTTTTTCTATGGAGAAATCTAGATCAGCATGATGTCTGACTAGACGCGTaagctctgcaactatttttagat |
| 5’ spl_UP | ttatcaggaaacagctatgaccgcggccgctattatagaaagttcatggg |
| 3’ spl_UP | atatagcaaatctttaggtaagatttgccatttatttcacctctacttaa |
| 5’ spl_DN | taaaaactttaagtagaggtgaaataaatggcaaatcttacctaaagatt |
| 3’ spl_DN | caggcttcttatttttatgctagctcgaggatttgctctatatttttctg |
| CRISPR_spl_647 | gtgtgctataattaaactgtaaaacgcgtgtcagctgtaaaacttgctaGTTTTAGAGCTAGAAATAGCAAGTTAAAATAAGGCTAGTCCGTTATCAACTTGAAAAAGTGGCACCGAGTCGGTGCTTTTTTTCTATGGAGAAATCTAGATCAGCATGATGTCTGACTAGACGCGTaagctctgcaactatttttagat |
| 5’ sspB UP | atttttttatcaggaaacagctatgaccgcggccgcttttaaaatatcatccatattat |
| 3’ sspB UP | tgtcaaaatttactatttattttccagccacctcaaataattagtttatgatg |
| 5’ sspB DN | tgttagacatcataaactaattatttgaggtggctggaaaataaatagta |
| 3’ sspB DN | tctgcgatcgcgcatgtctgcaggcctcgagatacttgtctattttttcagtaaca |
| CRISPR_sspB_144 | aattaaactgtaaaggtaccagagaaaatggatatgttggGTTTTAGAGCTAGAAATAGCAAGTTAAAATAAGGCTAGTCCGTTATCAACTTGAAAAAGTGGCACCGAGTCGGTGCTTTTTTTCTATGGAGAAATCTAGATCAGCATGATGTCTGACTAGACGCGTaagctctgcaacta |
| 5’ sspB.xylR | gtgttactgaaaaaatagacaagtatctcgagctagcataaaaataagaagcct |
| 5'CDR20291_1130_UP | atttttttatcaggaaacagctatgaccgcggccgcgcatttatcagaagatggt |
| 3'CDR20291_1130_UP | tttctcacatttaactttttttattccataagaatcctcctatcagtaaaatttat |
| 5'CDR20291_1130_DN | tactgataggaggattcttatggaataaaaaaagttaaatgtgagaaaataag |
| 3'CDR20291_1130_DN | tctgcgatcgcgcatgtctgcaggcctcgagatcaacaaatccttcaaca |
| CRISPR_CDR20291_1130_114 | gtgtgctataattaaactgtaaaacgcgttctaataaaggtgttttagaGTTTTAGAGCTAGAAATAGCAAGTTAAAATAAGGCTAGTCCGTTATCAACTTGAAAAAGTGGCACCGAGTCGGTGCTTTTTTTCTATGGAGAAATCTAGATCAGCATGATGTCTGACTAGACGCGTaagctctgcaactatttttagat |
| 5'CDR20291_3080_UP | atttttttatcaggaaacagctatgaccgcggccgcattttgccacatatacgctatat |
| 3'CDR20291_3080_UP | ttaattgacaaaaatacaaataagaagggggtgattttgcgtttgtatatg |
| 5'CDR20291_3080_DN | cataagttcatcatatacaaacgcaaaatcacccccttcttatttgtatttttgtc |
| 3'CDR20291_3080_DN | tctgcgatcgcgcatgtctgcaggcctcgagcagaagtgtatgagaaaatgaag |
| CRISPR_CDR20291_3080_184 | gtgtgctataattaaactgtaaaacgcgtTGCTTACATCACGCAAAAAGGTTTTAGAGCTAGAAATAGCAAGTTAAAATAAGGCTAGTCCGTTATCAACTTGAAAAAGTGGCACCGAGTCGGTGCTTTTTTTCTATGGAGAAATCTAGATCAGCATGATGTCTGACTAGACGCGTaagctctgcaactatttttagat |
| 3’cas9_Pxyl2 | taatcctatactatattttttatccatttaattaactctcctctttaccctcctt |
| 5’CD1130_HR_xylR | agctggaagtgttgaaggatttgttgatctcgagctagcataaaaataagaagcct |
| 5’CD3080_HR_xylR | ctctaagcttcattttctcatacacttctgctcgagctagcataaaaataagaagcct |
| 3’ sspA.pJS116 | tgccaagcttgcatgtctgcaggcctcgagctatctgttgctttttccag |
| 3’sspBpJS116 | cagtgccaagcttgcatgtctgcaggcctcgagttattttccagccatttgtc |
| 3’ PsspB_sspA | gttctgttgttgttattgttacttgccatataattagtttatgatgtcta |
| 5’ PsspB_sspA | agtgttgttagacatcataaactaattatatggcaagtaacaataacaac |
| 3’sspB_sspA | ttgaatttgacatagtaaatttcctcctcaagttgattaccttccttctg |
| 5’sspB_sspA | ataaattaaacagaaggaaggtaatcaacttgaggaggaaatttactatg |
| 5' 1130comp | ttatcaggaaacagctatgaccgcggccgcaaagcatttatcagaagatg |
| 3' 1130comp | gccaagcttgcatgtctgcaggcctcgagttattctaaatgcctagatatacc |
| 5' 3080comp | ttatcaggaaacagctatgaccgcggccgccccagaagtgtatgagaaaa |
| 3' 3080comp | caagcttgcatgtctgcaggcctcgagctatattgactcatcctttttattc |
| 3’ sspA_CD1130 | tagcattttgtcttgaaacatcatccatgttgattaccttccttctgttta |
| 5’ CD1130_sspA | ataaattaaacagaaggaaggtaatcaacatggatgatgtttcaagacaaaa |
| 3’ sspA_CD3080 | tttctctttgtactttttctttcaaaaaaatcacgttgattaccttccttctgtt |
| 5’ CD3080_sspA | aataaattaaacagaaggaaggtaatcaacgtgatttttttgaaagaaaaagtac |
| 3' PsspA.mCherry | gccatattatcttcttctcctttagataccatgttgattaccttccttctg |
| 5' PsspA.mCherry | taaattaaacagaaggaaggtaatcaacatggtatctaaaggagaagaag |
| 3' mCherry.PsspA | gtgccaagcttgcatgtctgcaggcctcgagttaaaacttataggatccgg |
| 3’ PsspA.pJS116 | agtgccaagcttgcatgtctgcaggcctcgaggttgattaccttccttc |
| 5’ sigE.bclA2_pJS116 | tttatcaggaaacagctatgaccgcggccgcttagtgccagattattgtgg |
| 3’ sigE.bclA2_sspB | tttgacatagtaaatttcctcctcaaataattaatcctccttttttaaag |
| 5’ sigE.bclA2_sspB | ctctaactttaaaaaaggaggattaattatttgaggaggaaatttactatg |
| 3’ sspB_pJS116 | tgccaagcttgcatgtctgcaggcctcgagttattttccagccatttgtc |
| 5’ pJS116_sigK | ttttatcaggaaacagctatgaccgcggccgctcagagcttttacaccttct |
| 3’ sigK_sspB | aatttgacatagtaaatttcctcctcaacaaatcaccctttctttaaatg |
| 5’ sigK_sspB | ctaaattcatttaaagaaagggtgatttgttgaggaggaaatttactatg |
| 5’ pJS116_sigF | ttatcaggaaacagctatgaccgcggccgcaagaaatgaagtgcgttaa |
| 3’ sigF_sspB | atttgacatagtaaatttcctcctcaaataaacctccagtataaattaaatg |
| 5’ sigF_sspB | attagcatttaatttatactggaggtttatttgaggaggaaatttactatg |
| 5’sspApJS116 | cgaattcgagctcggtacccggggatcctctagattagatgaggaaaaactggataaaag |
| 3’ sspAsspB | ggaactgataatatggatgatattttaaaactatctgttgctttttccagccatttg |
| 5’ sspAsspB | caaatggctggaaaaagcaacagatagttttaaaatatcatccatattat |
| 3’ sspA.His_pJS116 | tgcaggcctcgagctagtggtggtggtggtggtgtctgttgctttttccagc |
| 5' rpoA | taaaggtagaggttatgtttctgct |
| 3' rpoA | tttgaccaactcttgtgttttcc |
| 5' sspA_qPCR | caaaagaggctttaaaccaaatgaa |
| 3' sspA_qPCR | attttctcttgcagtaaggtttcctt |
| 5' sspB_qPCR | aacagaacagtagttccagaagcaaa |
| 3' sspB_qPCR | caacatatccattttctctagctgttaag |
| 5' CD3080_qPCR | cgtggatgggcaggactt |
| 3' CD3080_qPCR | cgtgatgtaagcatacctccaattc |
| 5'sleC_qPCR | ttgaagcaagacaaggagttccc |
| 3'sleC_qPCR | cgaaaccagtaggaggaggtaatgg |
| 5' spoVT_qPCR | agagaaggagaccctttagagat |
| 3' spoVT_qPCR | ctgttatcaacactccatatcctagt |
| 5' pdaA_qPCR | tggtaaacagccatcacctataa |
| 3' pdaA_qPCR | tccactttcatatccagcatca |
| 5'spoIVA_qPCR | ggatagaacaagagatgagataccc |
| 3'spoIVA_qPCR | ctgctgccttttcaaatgtc |
