# Supplementary material for: The small acid-soluble proteins of Clostridioides difficile are important for UV resistance and serve as a check point for sporulation
Source: PLoS Pathog. 2021 Sep 8;17(9):e1009516. doi: 10.1371/journal.ppat.1009516 (PMC8452069; doi:10.1371/journal.ppat.1009516)
Supplement: S2 Table — (DOCX) [file ppat.1009516.s008.docx]

**S2 Table. Strains and plasmids used in this study.**

| **Strain** | **Description** | **Reference** |
| --- | --- | --- |
| *E. coli* DH5a | F^-^ endA1 glnV44 thi-1 recA1 relA1 gyrA96 deoR nupGΦ80d*lacZ*ΔM15 Δ(*lacZ*YA-*argF*)U169, hsdR17(r_K_^-^m_K_^+^), λ- | [1] |
| *E. coli* MB3436 | *recA^+^ E. coli* strain | Gift from Dr. Michael Benedik |
| *B. subtilis* BS49 | *Tn*916 donor strain, Tet^R^ | [2] |
| *C. difficile* R20291 | Wild type, ribotype 027 | [3] |
| *C. difficile* KNM10 | *spo0A* CRISPR-*cas9* mutant | [4] |
| *C. difficile* HNN03 | *sspA* CRISPR-*cas9* mutant | This study |
| *C. difficile* HNN04 | *sspB* CRISPR-*cas9* mutant with a *sspA*_G52V_ allele | This study |
| *C. difficile* HNN05 | *sspA* and *sspB* CRISPR-*cas9* double mutant | This study |
| *C. difficile* HNN06 | *CDR20291_1130* CRISPR-*cas9* mutant | This study |
| *C. difficile* HNN07 | *CDR20291_3080* CRISPR-*cas9* mutant | This study |
| *C. difficile* HNN10 | *spl* CRISPR-*cas9* mutant | This study |
| *C. difficile* HNN11 | *sspA* and *spl* CRISPR-*cas9* double mutant | This study |
| *C. difficile* HNN12 | *CDR20291_1130* and *CDR20291_3080* CRISPR-*cas9* double mutant | This study |
| *C. difficile* HNN14 | *sspA* and *CDR20291_3080* CRISPR-*cas9* double mutant | This study |
| *C. difficile* HNN15 | *sspA* and *CDR20291_1130* CRISPR-*cas9* double mutant | This study |
| *C. difficile* HNN16 | *sspA* and *CDR20291_1130* and *CDR20291_3080* CRISPR-*cas9* triple mutant | This study |
| *C. difficile* HNN17 | *sspB* CRISPR-*cas9* mutant | This study |
|  |  |  |
| **Plasmid** | **Description** | **Reference** |
| pJS116 | *B. subtilis* – *C. difficile* shuttle vector pCD6 ColE1 *Tn916 oriT* Cm^R^ | [5] |
| pKM126 | CRISPR plasmid with *tetR* promoter driving *cas9* | [6] |
| pKM197 | CRISPR plasmid with *xylR* promoter driving *cas9* | [7] |
| pCE641 | *xylR* containing plasmid | [8] |
| pRAN473 | *mCherry* containing plasmid | [9] |
| pGC05 | *sspB* targeting CRISPR plasmid | This study |
| pHN05 | *sspA* targeting CRISPR plasmid | This study |
| pHN11 | *sspA* promoter region and gene | This study |
| pHN14 | *sspB* promoter region and gene | This study |
| pHN30 | *sspA* and *sspB* complement | This study |
| pHN32 | *1130* targeting CRISPR plasmid | This study |
| pHN34 | *3080* targeting CRISPR plasmid | This study |
| pHN47 | *gpr* promoter region and *sspB* gene | This study |
| pHN49 | *sleC* promoter region and *sspB* gene | This study |
| pHN56 | *1130* promoter region and gene | This study |
| pHN57 | *3080* promoter region and gene | This study |
| pHN61 | *spl* targeting CRISPR plasmid | This study |
| pHN80 | *bclA2* promoter region and *sspB* gene | This study |
| pHN83 | *sspA* promoter region and *sspB* gene | This study |
| pHN84 | *sspA* promoter region and gene with 6x His tag on the C-terminus | This study |
| pHN91 | *sspB* promoter and *sspA* gene | This study |
| pHN96 | *sspA* promoter region and *1130* gene | This study |
| pHN97 | *sspA* promoter region and *3080* gene | This study |
| pHN101 | *sspB* targeting CRISPR plasmid, *xylR* promoter | This study |
| pHN102 | *sspA* promoter region | This study |
| pHN109 | *sspA* promoter region and *mCherry* gene | This study |
| pHN118 | *sspA*_G52V_ allele and promoter | This study |

**Supplemental Information References**

1. Hanahan D. Studies on transformation of *Escherichia coli* with plasmids. J Mol Biol. 1983;166(4):557-80. Epub 1983/06/05. PubMed PMID: 6345791.

2. Bouillaut L, McBride SM, Sorg JA. Genetic manipulation of *Clostridium difficile*. Curr Protoc Microbiol. 2011;Chapter 9:Unit 9A 2. doi: 10.1002/9780471729259.mc09a02s20. PubMed PMID: 21400677; PubMed Central PMCID: PMC3615975.

3. Stabler RA, He M, Dawson L, Martin M, Valiente E, Corton C, et al. Comparative genome and phenotypic analysis of *Clostridium difficile* 027 strains provides insight into the evolution of a hypervirulent bacterium. Genome Biol. 2009;10(9):R102. Epub 2009/09/29. doi: 10.1186/gb-2009-10-9-r102. PubMed PMID: 19781061; PubMed Central PMCID: PMCPMC2768977.

4. McAllister KN, Martinez Aguirre A, Sorg JA. The selenophosphate synthetase, *selD*, is important for *Clostridioides difficile* physiology. J Bacteriol. 2021. Epub 2021/04/07. doi: 10.1128/JB.00008-21. PubMed PMID: 33820795.

5. Sorg JA, Sonenshein AL. Bile salts and glycine as cogerminants for *Clostridium difficile* spores. J Bacteriol. 2008;190(7):2505-12. doi: 10.1128/JB.01765-07. PubMed PMID: 18245298; PubMed Central PMCID: PMCPMC2293200.

6. McAllister KN, Bouillaut L, Kahn JN, Self WT, Sorg JA. Using CRISPR-Cas9-mediated genome editing to generate *C. difficile* mutants defective in selenoproteins synthesis. Scientific Reports. 2017;7(1):14672. doi: 10.1038/s41598-017-15236-5.

7. Bhattacharjee D, Sorg JA. Factors and conditions that impact electroporation of *Clostridioides difficile* strains. mSphere. 2020;5(2). Epub 2020/03/07. doi: 10.1128/mSphere.00941-19. PubMed PMID: 32132157; PubMed Central PMCID: PMCPMC7056809.

8. Kaus GM, Snyder LF, Muh U, Flores MJ, Popham DL, Ellermeier CD. Lysozyme resistance in *Clostridioides difficile* is dependent on two peptidoglycan deacetylases. J Bacteriol. 2020;202(22). Epub 2020/09/02. doi: 10.1128/JB.00421-20. PubMed PMID: 32868404; PubMed Central PMCID: PMCPMC7585060.

9. Ransom EM, Ellermeier CD, Weiss DS. Use of mCherry Red fluorescent protein for studies of protein localization and gene expression in *Clostridium difficile*. Appl Environ Microbiol. 2015;81(5):1652-60. Epub 2014/12/21. doi: 10.1128/AEM.03446-14. PubMed PMID: 25527559; PubMed Central PMCID: PMCPMC4325159.
